# Autologous Stem Cell Transplantation for Chronic Lymphocytic Leukemia - Still a Valid Treatment Option, or is the Game Over?

**DOI:** 10.4084/MJHID.2012.071

**Published:** 2012-11-06

**Authors:** Fabienne McClanahan, Peter Dreger

**Affiliations:** 1 Department of Medicine V, University of Heidelberg, Heidelberg, Germany; 2 Centre for Haemato-Oncology, Barts Cancer Institute, Queen Mary, University of London, London, UK

## Abstract

Chemoimmunotherapy with fludarabine, cyclophosphamide, and rituximab (FCR) has been established as the current standard of care for young and fit patients with chronic lymphocytic leukemia (CLL). In the early nineties of the last century, long before the advent of fludarabine or antibody-based strategies, there was realistic hope that myeloablative therapy followed by autologous stem cell transplantation (autoSCT) might be an effective and potentially curative front-line treatment option for suitable patients with CLL. Since then, several prospective trials have disenthralled this hope: although autoSCT can prolong event and progression-free survival if used as part of early front-line treatment, it does not improve overall survival, while it is associated with an increased risk of late adverse events such as secondary malignancies. In addition, autoSCT lacks the potential to overcome the negative impact of biomarkers that confer resistance to chemotherapy or early relapse. The role of autoSCT has also been explored in the context of FCR, and it was demonstrated that its effect is inferior to the currently established optimal treatment regimen. In view of ongoing attempts to improve on FCR, promising clinical activity of new substances even in relapsed/ refractory CLL patients, exciting novel cell therapy approaches and advantages in the understanding of the disease and detection of Minimal Residual Disease (MRD), autoSCT has lost its place as a standard treatment option for CLL.

## Introduction

Chronic lymphocytic leukemia (CLL) is one of the most common lymphoid malignancies,^1^ and the most common adult leukemia in Western countries.^1^,^2^ Large multicenter trials have established the combination chemoimmunotherapy of fludarabine, cyclophosphamide, and rituximab (FCR) as the current standard of care for young patients without any concomitant diseases.^3^,^4^ This approach, however, is neither curative nor is it suited for the very elderly and those with comorbidities. In addition, there is a subgroup of high-risk patients which poorly respond to chemoimmunotherapy and suffer from early relapse.^5^ Therefore, there is a substantial need to explore alternative therapeutic approaches.

## Phase-II Trials on Autologous SCT in CLL

Long before the advent of FCR and other fludarabine or monoclonal antibody-based strategies, several studies suggested that autologous stem cell transplantation (autoSCT) might represent an effective and potentially curative treatment option for suitable patients with CLL.^6^–^10^ The 2005 update from the original Dana-Faber-Cancer-Institute (DFCI) single-center series showed a 6-year progression-free survival (PFS) of 30% and a 6-year overall survival (OS) of 58% after autoSCT.^11^ In the UK MRC pilot study, a multicenter phase-II trial on autoSCT as part of first-line CLL treatment, the 5-year OS and PFS rates were 78% and 52%.^12^ In 1996, the German CLL Study Group (GCLLSG) designed a large phase-II multicenter trial to assess the feasibility and efficacy of early autoSCT in poor-risk CLL. Compared to the UK study, the CLL3 trial followed a very aggressive treatment approach by including poor-risk patients at an early stage of disease (i.e. patients were lacking conventional treatment indications) and applying Dexa-BEAM as mobilization to improve mobilization efficacy and disease control before autoSCT (dose-identification). After a median follow-up of 8.7 years, median PFS and OS were 5.7 years and 11.3 years respectively.^13^ PFS was significantly affected by unfavourable IGHV (p < .001) and 17p- (p < .001) in a multivariate setting. Predictors of a shorter OS in a multivariate setting were 17p- (p < .001), unfavourable IGHV (p = .008) and Binet stage C (p = .03). Partly reflecting the high toxicity of this intense treatment regimen, the cumulative incidence of non-relapse-mortality (NRM) was 6.5% after 5 years and 14% after 10 years. Although these phase II trials indicated that autoSCT could - when used as part of first-line therapy -effectively control the disease in a subgroup of patients, their long-term follow-up data provided little evidence of curative potential in a substantial proportion of patients. Further details and the main findings of these phase II studies are summarised in table 1.

## Phase-III Trials on Autologous SCT in CLL

Subsequently, prospective randomized trials were opened for enrollment, and despite being initiated in the pre-Rituximab era, the results have been eagerly awaited for. The French intergroup trial randomized patients in complete remission (CR) after mini-CHOP-and fludarabine-based therapy to autoSCT or observation. Those patients who did not achieve CR, received cytarabine-based salvage therapy followed by autoSCT or FC.^14^ While autoSCT significantly increased 3-year event-free survival (EFS) in CR patients (80% vs. 36%, p = .003), there was no difference in EFS between the treatment groups in non-CR patients and in OS in all response subgroups. An EBMT phase-III multinational trial randomized patients in CR or PR after first- or second-line treatment to consolidating autoSCT or watchful waiting,^15^ including virtually all CR patients from the French phase III trial. While autoSCT almost doubled EFS and time to retreatment (TTRT), it did not have a significant impact on OS. A similar pattern was observed in the small French GOELAMS LLC 98 trial, which randomized patients (n=86) between conventional CHOP chemotherapy and CHOP followed by upfront autoSCT in remission:^16^ as observed in the previous randomized trials, autoSCT significantly prolonged PFS but was lacking any survival advantage. Table 2 summarizes the major findings of these phase III trials. However, the results from all phase III trials need to be interpreted with a certain degree of caution, as neither trial applied any upfront therapy that would be in accordance with today’s gold-standard treatment (i.e. FCR).

## Comparison autoSCT versus FCR

A direct prospective randomized comparison between Rituximab based therapy and auto-SCT has never been undertaken, and in the view of promising new agents and cellular therapy approaches, it can be assumed that this is very unlikely to happen in the future either. However, in a recent study within this setting, 110 patients from the GCLLSG autoSCT CLL3 trial and 126 patients from the FCR arm of the GCLLSG CLL8 trial were retrospectively compared.^13^ Patients were matched for age, time from diagnosis to study entry, serum thymidine kinase levels, cytogenetic risk group by fluorescence-in-situ-hybridisation (FISH) and IGHV mutation status. In this cohort, PFS was significantly longer in the autoSCT group (median 6.2 years) than in the FCR group (median 4.3 years), which however did not translate into prolonged TTRT (median 7.7 years vs. not reached, p=.91) as observed in the EBMT trial. Four-year OS (86% vs. 90%, p=.39) was comparable between autoSCT and FCR. Unfavorable IGHV status was the only factor significantly affecting TTRT in a multivariate setting, whereas OS was adversely influenced by unfavorable IGHV, Binet C stage and age. Specific poor-risk subgroups did not benefit from autoSCT. Although these results are probably the closest to a valid comparison between FCR and autoSCT, they should be interpreted with caution: all findings are based on a retrospective analysis, with the limitations of such an approach being frequently discussed. In addition, there were differences between the two patient populations that could not be eliminated by matching, especially the duration and intensity of follow-up.

## AutoSCT in Richter’s Syndrome

A recently published retrospective EBMT study suggested that autoSCT might play a role in the treatment of patients with chemo-sensitive Richter's syndrome.^17^ Although autoSCT seemed to lack a curative effect, the estimated probability of surviving 3 years after autoSCT was more than 50%, which compared favourably to the survival of chemotherapy-sensitive patients without autoSCT consolidation in another series.^18^

## Secondary Malignancies

In addition to the lack of full curative potential and convincing advantages over conventional chemoimmunotherapy, serious long-term effects such as solid tumours and secondary haematological malignancies need to be taken into consideration after autoSCT. The 2008 WHO-classification of haematopoietic and lymphoid tissues has implemented therapy-related myeloid neoplasms (t-MNs) as a separate category, which includes therapy-related acute myeloid leukaemia (t-AML), myelodysplastic syndrome (t-MDS) and myelodysplastic/ myeloproliferative neoplasms (t-MDS/MPN).^19^ T-MNs have emerged as serious long-term complications of cytotoxic therapy for CLL:^20^–^26^ among 2,028 patients with CLL/ small lymphocytic lymphoma (SLL) treated at the MD Anderson Cancer Centre from 1985 to 2005, 11% developed other malignancies during the follow-up period, and the risk of a second cancer was 2.2 times higher than the expected risk calculated from the SEER database.^21^ In contrast, a population-based analysis of the SEER database of 1-year survivors with CLL/ SLL (n=15,915), revealed that CLL patients have a significantly elevated risk for lung cancer and melanoma, but not for acute non-lymphocytic leukaemia when compared to other lymphoid malignancies.^27^ In a randomized study comparing treatment with chlorambucil, fludarabine, or fludarabine plus chlorambucil (FC), 1.2% developed t-MNs after a median follow-up of 4.2 years, with the majority occurring after combination FC therapy.^28^ In another study, 6% of CLL patients developed t-MNs 5 years after treatment with combination fludarabine.^20^ In a long-term follow-up study of first-line FCR, there were eight cases of MDS (2.8%) that occurred during first remission.^3^ In contrast, no case of t-MN was observed in the CALGB 9712 trial after fludarabine plus rituximab (FR) therapy.^29^ Long-term follow-up observations have raised concerns that the incidence of t-MNs might be even more pronounced after autoSCT: in a Finnish analysis of patients being treated with autoSCT from 1990 to 2003, the risk of NRM was highest in patients with CLL (9.5%), with another malignancy being the most common cause of late NRM.^30^ The most common forms of fatal secondary malignancies were t-MNs. In the DFCI and MRC series, t-MN occurred in 9% and 8% of patients, translating into a 5- and 8-year incidence-rate of 12%.^11^,^31^ In the CLL3 trial, the 10-year incidence was 19%, with no significant difference of any secondary malignancy among individuals treated with and without autoSCT.^13^ However, all cases of t-MN occurred after autoSCT, yielding a 10-year incidence rate of t-MN of 8%, which is in the range of the DFCI and MRC series. Within all series, the outcome after t-MNs was poor, which makes this a very relevant and serious long-term complication and is particularly relevant to patients that may have benefited from less aggressive regimens.

## Conclusions and Perspectives

What major conclusions can be drawn from almost two decades of clinical trials on autoSCT in CLL? Firstly, autoSCT indeed has the capacity to provide prolonged disease control at least similar to modern immunochemotherapy regimens, such as FCR. However, like immunochemotherapy, autoSCT has no significant curative potential in CLL. Secondly, autoSCT does not have the potential to overcome the negative impact of biomarkers that confer resistance to chemotherapy. Therefore, patients who have gained the highest benefit from autoSCT would also most likely respond to conventional immunochemotherapy. Thirdly, autoSCT is associated with an increased risk of secondary neoplasms, which is a very serious long-term adverse event with a poor outcome.

Over the past few years, CLL research and treatment have made a huge leap forward: to name a few examples, randomized clinical trials aiming to improve the potential of FCR and to optimize treatment for high-risk and older patients or those with comorbidities are ongoing, and their recruitment has for the most part been exceeding expectations. There are also several exciting new small molecule inhibitors such as the BTK-inhibitor ibrutinib or the bcl-2 inhibitor navitoclax, which show promising activity even in the relapsed/ refractory setting, and in combination with conventional chemoimmunotherapy.^32^–^34^ Several studies have indicated that allogeneic HSCT is currently the only treatment with curative potential on the basis of its capacity to induce graft-versus-leukemia (GVL) activity, even in high-risk CLL patients.^35^–^37^ This approach, however, is never indicated as part of first-line treatment in standard-risk patients and should be restricted to patients who meet the EBMT transplant consensus criteria.^38^ Sensitive techniques for MRD quantification have been further optimized and might serve as a surrogate marker to assess treatment efficacy.^39^ In addition, experimental treatment approaches such as chimeric-antigen-receptor (CAR) T cells have shown exciting preliminary results which lead us to believe that this might direct us into the future of CLL treatment.^40^ In view of these and other developments, autoSCT does currently not play a role in the treatment of CLL; therefore the autoSCT game indeed seems to be over for the time being.^41^

## Figures and Tables

**Table 1:** Overview of phase II trials on autoSCT in CLL.

	*DFCI (11)*	*MRC pilot trial (12)*	*GCLLSG CLL3 study (13)*
***Major patient eligibility criteria***	Binet stage B Poor risk Binet stage C Up to 66 years	1^st^-line treatment Binet stage B or C or progressive stage A Up to 60 years	1^st^-line treatment Binet stage B or C, poor risk Binet A 18–60 years

***Patients receiving auto SCT, n***	137	65	131

***Median age, years (range)***	49 (19–66)	49 (27–60)	51 (27–60)

***Stem cell source***	Bone marrow, B-cell depleted	Peripheral blood, CD34-selected	Peripheral blood, CD34-selected

***Study treatment***			
*Cytoreduction*	any	F/ FC/ Al/ CHOP	CHOP/ F/ FC
*Mobilization*	not applicable	F/ Cy	Dexa-BEAM
*Conditioning*	TBI 14Gy/ Cy	TBI 14 or 12Gy/ Cy or BEAM	TBI 12Gy/ Cy

***Duration of follow-up (years)***	6.5	3	8.7

***PFS or DFS (%)***	6-years: 30 (±4)	5 years: 51.5 (CI 33.2–69.8)	Median: 5.7 years

***OS (%)***	6 years: 58 (±5)	5 years: 77.5 (CI 57.2–97.8)	Median: 11.3 years

***Any 2nd NPL, n***	31	n.a.	20

***t-MN, n***	13	8	6

***Incidence (%), range***	8 years: 12 (5–19)	5 years: 12.4 (2.5–24)	10 years: Any 20 (11–30) t-MN 8 (0–16)

***Interval AutoSCT to t-MNS, mo (range)***	36 (11–87)	39 (17–73)	53 (8–86)

F = fludarabine, FC = fludarabine/ cyclophosphamide, Al = alemtuzumab, CHOP = cyclophosphamide, doxorubicine, vincristine, prednisone, Cy = cyclophosphamide, TBI = total body irradiation, Gy = Gray, BEAM = carmustin, etoposide, cytarabine, melphalan, PFS = progression-free-survival, DFS = disease-free-survival, CI = confidence interval, OS = overall survival, NPL = neoplasm, t-MN = therapy-related myeloid neoplasm, mo = months

**Table 2:** Phase III trials on autoSCT in CLL.

	*SFGM-TC/ GFLLC (14)*	*EBMT (15)*	*GOELAMS LLC 98 (16)*
***Randomisation arms***	After mini-CHOP/ F: autoSCT vs. observation in CR DHAP salvage plus autoSCT vs. FC in non-CR	After CR/PR after any first- or second-line treatment: autoSCT vs. w&w	CHOP-based vs. CHOP plus autoSCT in CR/very good PR
***Patients, n***	52 vs. 53 in CR* 46 vs. 48non-CR	112 vs. 111	39 vs. 43
***Median age, years (range)***	All: 56 (31–66)	54 (31–65) vs. 53 (35–65)	54 (35–60) vs. 55 (40–61)
***EFS/ PFS (%)***	At 3 years: CR pts: 80 (CI 69–92) vs. 35 (CI24–52) Non-CR pts: 49 (CI 35–68) vs. 44 (CI 32–62)	At 5 years: 42 vs. 24	Median: 22 mo (CI 13–31) vs. 53 mo (CI 40–66)
***OS (%)***	At 3 years: CR pts: 96 (CI 90–100) vs. 98 (CI 94–100) Non-CR pts: 82 (CI 71–94) vs. 87 (CI 78–97)	At 5 years: 86 vs. 84	Median: 105 mo (100–110) vs. 107 mo (CI 58–157)

CHOP = cyclophosphamide, doxorubicine, vincristine, prednisone, F = fludarabine, CR = complete remission, PR = partial remission, FC = fludarabine/ cyclophosphamide, w&w = watch and wait, EFS = event-free-survival, PFS = progression-free-survival, CI = confidence interval, OS = overall survival, mo = months.

* The CR population of the SFGM-TC trial was also part of the EBMT trial.
